# Expression of concern: Intrauterine insemination vs timed intercourse with clomiphene citrate in polycystic ovary syndrome: A randomized controlled trial

**DOI:** 10.1111/aogs.14846

**Published:** 2024-04-18

Hatem Abu Hashim, Osama Ombar, and Ibrahim Abd Elaal. (2011), Intrauterine insemination vs timed intercourse with clomiphene citrate in polycystic ovary syndrome: a randomized controlled trial. Acta Obstetricia et Gynecologica Scandinavica, 90: 344‐350. https://doi.org/10.1111/j.1600‐0412.2010.01063.x

This Expression of Concern is for the above article, published online on 03 January 2011, in Wiley Online Library (wileyonlinelibrary.com), and has been published by agreement between the journal's Editor‐in‐Chief, Ganesh Acharya, the Nordic Federation of Societies of Obstetrics and Gynecology, and John Wiley and Sons Ltd. The Expression of Concern has been agreed following information shared by Vrije Universiteit Brussel where one of the authors undertook their PhD. Concerns have been uncovered regarding the statistical analysis and the authenticity of the data. The authors disagree with the concerns raised. An institutional investigation at Mansoura University into the reliability of the published Clinical trial was requested in 2021. The author has previously provided a letter from Mansoura University, dated 17 March 2012, which confirms the author did work and publish studies between 2009 and 2012 at the institute. However, we were unsuccessful in receiving a direct response from Mansoura University with regard to the current investigation despite our attempts. The journal has decided to issue an Expression of Concern to alert the readers as the authenticity of the data could not be confirmed.

